# Preventing psychosis in people at clinical high risk: an updated meta-analysis by the World Psychiatric Association Preventive Psychiatry section

**DOI:** 10.1038/s41380-025-02902-8

**Published:** 2025-02-14

**Authors:** Amedeo Minichino, Cathy Davies, Olga Karpenko, Nikos Christodoulou, Rodrigo Ramalho, Sunil Nandha, Stefano Damiani, Umberto Provenzani, Cecilia Maria Esposito, Martina Maria Mensi, Renato Borgatti, Alberto Stefana, Philip McGuire, Paolo Fusar-Poli

**Affiliations:** 1 Department of Psychiatry, University of Oxford, Oxford, UK; 2 EPIC Lab, Department of Psychosis Studies, Institute of Psychiatry, Psychology and Neuroscience, King’s College London, London, UK; 3 Mental-health Clinic No. 1 named after N.A. Alexeev, Moscow, Russia; 4 Department of Psychiatry, Faculty of Medicine, University of Thessaly, Larisa, Greece; 5 Department of Social and Community Health, School of Population Health, University of Auckland, Auckland, New Zealand; 6 Outreach And Support in South London (OASIS) Service, South London & Maudsley NHS Foundation Trust, London, UK; 7 Dipartimento di Scienze del Sistema Nervoso e del Comportamento, Università di Pavia, Pavia, Italy; 8 Child and Adolescent Neuropsichiatry Unit, IRCCS F. Mondino, Pavia, Italia; 9 Department of Psychiatry and Psychotherapy, University Hospital, Ludwig-Maximilian-University (LMU), Munich, Germany; 10 Present Address: Child and Adolescent Neuropsichiatry Unit, IRCCS F. Mondino, Pavia, Italia

**Keywords:** Schizophrenia, Depression

## Abstract

Recently published large-scale randomised controlled trials (RCTs) have questioned the efficacy of preventive interventions in individuals at clinical high risk for psychosis (CHR-P). We conducted a systematic review and meta-analysis to include this new evidence and provide future directions for the field. We followed the PRISMA guidelines and a pre-registered protocol, with a literature search conducted from inception to November 2023. We included RCTs that collected data on psychosis transition (the primary outcome) in CHR-P. Secondary outcomes were symptoms severity and functioning. Investigated time points were 6,12,24,36, and +36 months. We used odd ratios (ORs) and standardised mean differences (SMD) as summary outcomes. Heterogeneity was estimated with the Higgins I^2^. Twenty-four RCTs, involving 3236 CHR-P individuals, were included. Active interventions were Cognitive Behavioural Therapy (CBT), family-focused therapy, Integrated Psychological Therapy, antipsychotics, omega-3 fatty acids, CBT plus risperidone, minocycline, and other non-pharmacological approaches (cognitive remediation, sleep-targeted therapy, brain stimulation). Results showed no evidence that any of the investigated active interventions had a sustained and robust effect on any of the investigated outcomes in CHR-P, when compared to control interventions, including CBT on transition to psychosis at 12 months (9 RCTs; OR: 0.64; 95% CI: 0.39–1.06; I^2^: 21%; *P* = 0.08). These results highlight the need for novel treatment approaches in CHR-P. Future studies should consider the heterogeneity of this clinical population and prioritise stratification strategies and bespoke treatments.

## Introduction

Psychotic disorders are a leading cause of disability globally [1]. Early interventions in people with First-Episode Psychosis (FEP) can reduce the severity and progression of illness [2, 3]. Intervening before the onset of FEP has the potential to further optimize the benefits of early intervention [4–6]. The clinical high risk for psychosis (CHR-P) paradigm, based on subclinical symptoms of psychosis, is one of the most established preventive approaches in psychiatry [4–8]. Individuals that meet CHR-P criteria are typically detected in specialised preventive clinics located in the community [9–11], and assessed with specific psychometric interviews, which have excellent prognostic accuracy, at least in terms of group-level predictions [12].

Transition risk from a CHR-P state to psychosis varies over time, cumulating to 0.20 (95% CI, 0.19–0.21; *N* = 2357) at 2 years and 0.35 (95% CI, 0.32–0.38; *N* = 114) at 10 years [13]. This risk is variable (I^2^ from 77.9 to 95.8%), stratified across CHR-P subgroups [14], and highest in CHR-P individuals presenting with a short-lived psychotic episode [14–21]. The prevalence of CHR-P features is about ten times higher in clinical samples (19.2%) than in the general population (1.7%) [22]. A recent meta-umbrella systematic review showed that effective interventions in CHR-P could prevent up to 12.4% of all cases of psychosis [23].

However, a network meta-analysis found a lack of evidence to favour any active intervention for prevention of psychosis needs-based care [24, 25], findings that were replicated by an independent pairwise meta-analysis [26]. Since then, several new randomised controlled trials (RCTs) have reported results from a range of different preventive approaches in CHR-P samples [27–31]. We therefore conducted an updated systematic review and meta-analysis on preventive interventions for CHR-P individuals that incorporated the entire literature to date.

## Methods

This systematic review and meta-analysis followed an a priori protocol (Registration 10.17605/OSF.IO/EUSKB). The AMSTAR-2 checklist [32] and a list of changes from the protocol, are provided in the supplement.

### Included interventions

Based on our previous work [25] and on a preliminary search of the literature, we identified the following types of preventative interventions: (1) Cognitive Behavioural Therapy (CBT); (2) Family-focused therapy; (3) Integrated Psychological Therapy (i.e., CBT plus Cognitive remediation plus Psychoeducation); (4) Other psychotherapy or psychosocial approaches (e.g., ‘systemic therapy’ or sleep-targeted therapy); (5) Cognitive remediation; (6) Omega-3 fatty acids; (7) Antipsychotics; (8) CBT plus antipsychotics; (9) transcranial magnetic brain stimulation (TMS).

Depending on the active treatment tested in the individual RCTs, control interventions spanned from needs based interventions (NBI, as defined in [33]) to placebo pills, computer game/non-specific computer tasks, and sham TMS.

### Search strategy and selection criteria

An electronic search was conducted on MEDLINE and Web of Science from inception to the 28th of November 2023 using the following keywords: (risk OR prodromal OR prodrom* OR ultra-high risk OR clinical high risk OR high risk OR genetic high risk OR at risk mental state OR risk of progression OR progression to first-episode OR prodromally symptomatic OR basic symptoms) AND (psychosis) AND (RCT OR randomized controlled trial OR placebo controlled trial OR trial).

To be included in our systematic review, studies had to meet the following criteria: randomised controlled trials (RCTs, including cluster-randomised and cross-over trials) conducted in CHR-P individuals defined as below; original studies (articles, conference abstracts, reports on trial registries) written in any language; studies designed to be blinded (single or double-blind), even if blinding was not maintained during the course of the trials (as this often occurs in trials testing psychotherapy/psychosocial interventions); including data on psychosis transition (our primary outcome).CHR-P individuals had to be defined with recognised assessment instruments for CHR-P ascertainment, such as the Comprehensive Assessment of At-Risk Mental States (CAARMS) [7], the Structured Interview for Psychosis-risk Syndrome (SIPS) [34] and related Brief Intermittent Psychotic Symptom syndrome (BIPS), the Brief Psychiatric Rating Scale (BPRS) [35], the Positive And Negative Syndrome Scale (PANSS) [36], the Early Recognition Inventory (ERIraos) [37], the Bonn Scale for the Assessment of Basic Symptoms (BSABS) [38], the Basel Screening Instrument for Psychosis (BSIP) [39], the Schizophrenia Proneness Instrument - Adult (SPI-A) and Child and Youth (SPI-CY) version [40]. No restrictions were set on participants’ gender, concomitant medications (e.g., anxiolytics, antidepressants), and recruitment methods.

We excluded: studies without a control intervention; unblinded studies; quasi-randomised trials; studies that included participants under the age of 12; studies with a sample size less than 10 per arm; studies where participants were not randomised but only stratified on a variable (e.g., into locally determined high vs. relatively low risk groups within the CHR designation) and allocated to a treatment/control arm based on this factor; unpublished literature; studies that did not collect data on transition to psychosis. In case of articles presenting data on overlapping samples, we used the most recent data or the data with the largest sample size.

In line with PRISMA guidelines, the literature search and the study selection were conducted by two independent reviewers.

### Outcome measures and data extraction

Our primary outcome was transition to a FEP from a CHR-P state. From each study, we extracted the raw number of individuals that experienced an FEP from the active and control arms. These numbers were used to calculate an odds ratio (OR), with the total number of those randomised to each arm as the denominators. Transitions were counted as defined by the study-specific primary outcome measure for counting transitions.

Secondary outcomes were: (i) acceptability of intervention (discontinuation due to any cause), measured as the number of participants who dropped out of each arm for any reason following randomization; (ii) attenuated psychotic symptoms (APS) severity, measured as the overall score of positive symptom subscales of either the CAARMS, the SIPS, the SOPS, the BIPS, the BPRS, the PANSS, or the ERIraos; (iii) negative symptom severity, as measured by either the CAARMS, the SIPS, the BIPS, the BPRS, the PANSS, the ERIraos, or Scale for the Assessment of Negative Symptoms (SANS) [41]; (iv) functioning, as measured by the Global Assessment of Functioning (GAF) [42], the Social and Occupational Functioning Assessment Scale (SOFAS)[43], or the Global Functioning: Social and Role scales (GF:S and GF:R) [44].

For secondary outcomes, we extracted end of follow-up (endpoint) data. When endpoint data were available, we used change scores (from baseline to end of follow-up). Due to the variable effect of time on clinical outcomes in CHR-P, each outcome was assessed separately at discrete time points 6, 12, 18, 24, 36 and >36 months’ (if sufficient data were available). For studies where the final follow-up data fell within a temporal range (e.g., 11 months – temporal range 6 to 12 months), data were included in the analysis of the closest discrete time point (i.e., 12 months following our previous example).

In line with PRISMA guidelines, data extraction was conducted by two independent reviewers. When data required for meta-analysis was unreported, we contacted corresponding authors to request additional data. In case of non-response, data were extracted from graphs using a validated procedure [45].

### Strategy for data synthesis

Individual meta-analyses were conducted when two or more studies for the same intervention were available. All meta-analyses were conducted in R, version 4.2.3, using the *metabin* and *metacont* functions of the *meta* package. The standardized mean difference (SMD) and OR were used as summary statistics. Between-study heterogeneity was assessed by calculating Higgins I^2^ based on Cochrane Q indexes.

To assess the robustness of the results, we performed sensitivity analyses by sequentially removing single studies and rerunning the analysis.

### Risk of bias and publication bias

We used the Risk of Bias two tool from the Cochrane Handbook for Systematic Reviews of Interventions [46]. RCTs were classified as at high risk of bias if the trial was judged to be at high risk of bias in at least one domain (randomisation process, deviations from the intended interventions, missing outcome data, measurements of outcome, selection of reported results) or to have some concerns in at least three domains and/or in a way that substantially lowered confidence in the results (see also Supplementary material).

The presence of publication bias was assessed by visual inspections of funnel plots.

## Results

### Database

**Fig. 1:**
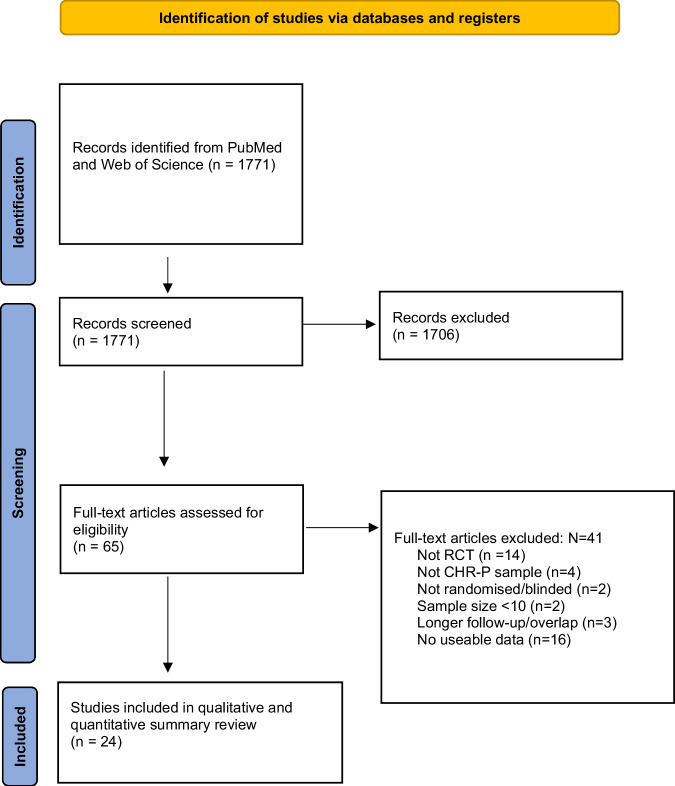
Prisma flowchart.

A total of 24 RCTs [27–31, 47–72] were included in our synthesis (see Fig. 1, PRISMA flowchart). This related to a total of 3236 CHR-P, with an average age of 19.9 ± 4.1 and a proportion of females ranging from 65 to 26%. The studies were representing different continents: Europe (*N* = 8, 35%), North America (*N* = 7, 30%), Australia (*N* = 6, 26%), and Asia (*N* = 3, 9%).

In these 24 RCTs, active interventions included: CBT in 10 RCTs with a total sample size of 1594 CHR-P [27–29, 47–54, 68]; family-focused interventions in 2 RCTs and 229 CHR-P [55, 56]; a sleep intervention in one RCT and 40 CHR-P [31]; a ‘systemic therapy’ approach in one RCT and 26 CHR-P [57]; cognitive remediation in one RCT and 146 CHR-P [58]; an integrated psychotherapy approach (i.e., CBT, cognitive remediation, and psychoeducation) in one RCT and 128 CHR-P [59]. Control interventions for these trials included case management and supportive therapy (see Table 1). In two RCTs [53, 54, 67], the active intervention was a combined approach of CBT plus risperidone, compared to supportive therapy plus placebo pills (total sample size, 130 CHR-P). In four RCTs [28, 53, 54, 60, 61] the active intervention was antipsychotics alone (total sample size, 512 CHR-P), in four RCTs was ω-3 PUFAs [62–66, 71] (*N* = 676 CHR-P); in one RCT was minocycline [71] (*N* = 164 CHR-P); in one RCT was minocycline plus ω-3 PUFAs [71] (*N* = 164 CHR-P); the control intervention for these trials was placebo pills. Finally, one trial investigated active transcranial magnetic stimulation (TMS) compared to sham TMS [30].

**Table 1:** Qualitative summary of the included trials.

Cognitive Behavioural Therapy (CBT)													
Article (Name of trial, if any)	Country	Primary outcome measure for transition to psychosis	CHR-P Ascertainment method	Active Intervention – (description)	Control Intervention (description)	Duration of intervention	Assessment time points of interest for meta-anlysis	N Interv	Age Interv	Gender Interv Males N (%)	N Control	Age Control	Gender Control Males
1. Addington [47]	Canada	POPS (McGlashan [60])	SIPS	CBT	Supportive Therapy (Active listening supporting individuals to cope with current problems)	6 months	6, 12, 18, 24	27	20.8 (4.5)	18 (35.3)	24	21.1 (3.7)	18 (35.3)
2. Addington [29]	Canada/US	SIPS	SIPS	CBT + SST (Group CBT enriched with social skill training)	Supportive Therapy (Active listening supporting individuals to cope with current problems)	4.5 months	12	104	17.4 (4.0)	29 (41.4)	99	17.5 (4.1)	40 (48.8)
3. Bechdolf [28] (PREVENT)^a^	Germany	POPS	COGDIS / SIPS	CBT	Case Management + Placebo pill (Case Management: Psychoeducation on the at-risk mental state and pharmacotherapy)	12 months	6, 12	129	24.2 (5.4)	78 (60.5)	55	24.9 (5.4)	33 (60.0)
4. McGorry [27], (SMART)	Australia			CBT + Case Management	SPS (“Manualized supportive counselling and problem-solving strategy)	12 months	6	153	17.7* (3.1) *overall group	144* (42.1) *overall group	159	17.7* (3.1) *overall group	144* (42.1) *overall group
5. Morrison [48], [68] (EDIE)	UK	PANSS	PANSS	CBT + Case Management	Case Management (“to resolve crisis regarding social issues and mental health risks”)	6 months	6, 12, 36+	37	20.6 (4.9)	21 (60.0)	23	21.5 (5.2)	19 (82.6)
6. Morrison [49]	UK	CAARMS	CAARMS	CBT + Monitoring of mental state	Monitoring of mental state (Supportive listening + signposting to local services for unmet needs or crisis management)	6 months	6,12,18,24	144	20.7 (4.2)	89 (61.8)	144	20.7 (4.5)	91 (63.2)
7, Pozza [50]	Italy	SCID-I	CAARMS	CBT (CBT enriched with social skill training and interventions targeting comorbid anxiety/depression)	“TAU” (Supportive listening)	7 months	6, 14	29	25.4 (6.1)	19 (65.5)	29	26.0 (5.7)	17 (69)
8. Stain [51] (DEPth)	Australia	CAARMS	CAARMS	CBT	NDRL (Supportive listening)	6 months	6, 12	30	16.2 (2.7)	10 (33.0)	27	16.5 (3.2)	14 (48.0)
9. Van der Gaag [52], Ising [72] (EDIE-NL)	Netherlands	CAARMS	CAARMS	CBT (CBT + psychoeducation on dopamine supersensitivy)	“TAU” (“Treatment for the mental problems that they are seeking help for – e.g., depression, anxiety, ADHD”)	6 months	6,12,18,36+	98^b^	22.9 (5.6)	49 (50.0)	103	22.6 (5.5)	50 (48.5)
10. Yung [53]^a^, McGorry^a^ [54]	Australia	CAARMS	CAARMS	CBT + Placebo pill	Supportive therapy + Placebo pill (“Therapy aimed to provide the patient with emotional and social support, basic problem solving, stress management, psychoeducation about psychosis”)	12 months	6, 12	44	18 (2.7)	17 (39.0)	28	18.8 (3.7)	13 (46.0)
Family interventions													
1. McFarlane [55] (EDIP)	US	SIPS/POPS	SIPS	F-ACT (Family psychoeducation, assertive community treatment, supported education/employment, psychotropic medications)	Enhanced Treatment (Psychotropic drugs, individual case management, family education, crisis intervention)	Unclear	Up to 60 months	50	16.5 (3.1)	26 (52.0)	50	16.1 (2.8)	26 (52.0)
2. Miklovitz [56]	US	SIPS	SIPS	FFT-CHR (Family psychoeducation targeting stressors that may contribute to psychotic symptoms + social skill training)	Enhanced Care (Family educational intervention for 1 month)	6 months	6	66	17.3 (4.2)	39 (57.4)	63	17.4 (3.9)	35 (55.6)
Sleep Interventions													
1. Waite [31], (SleepWell)	UK	CAARMS	CAARMS	SleepWell (“Psychological intervention targeting three mechanisms that regulate sleep: sleep pressure, circadian rhythm, and hyper arousal”)	TAU (“Infrequent contact with a general practitioner for assessment, and prescription of psychotropic medication as needed”)	4 months	6, 12* *9 months	21	17.0 (2.2)	9 (42.0)	19	16.8 (2.8)	10 (48.0)
Systemic Therapy													
1. Shi [57]	China	SIPS	SIPS	ST (Systemic Therapy: “contextualise attenuated psychotic symptoms by addressing an individual’s social system to which he/she attaches importance”)	Supportive Therapy (Supportive listening)	6 months	6	13	18.9 (3.2)	4 (35.8)	13	18.9 (4.3)	8 (61.5)
Cognitive Remediation													
1. Glenthøj [58], (FOCUS)	Denmark	CAARMS	CAARMS	Cognitive remediation (Manualised neuro- and social-cognitive remediation)	TAU (“Regular contact with health professionals and supportive counselling”)	5 months	6	73	23.9 (4.7)	35 (48.0)	73	23.9 (3.8)	29 (39.8)
Integrated Psychotherapy (CBT + cognitive remediation)													
1. Bechdolf [59] (EIPS)	Germany	PANSS	ERIaos	IPI (CBT + SST+ cognitive remediation +family psychoeducation)	Supportive Counselling (Basic psychoeducation + active listening)	12 months	12, 24	63	25.2 (5.4)	39 (61.9)	65	26.8 (6.2)	42 (64.6)
Antipsychotics													
1. Bechdolf [28] (PREVENT)^c^	Germany	POPS	COGDIS /SIPS	Aripiprazole (5–15 mg/day) + Case Management (Psychoeducation on the at-risk mental state and pharmacotherapy)	Placebo pill + Case Management (Psychoeducation on the at-risk mental state and pharmacotherapy)	12 months	6, 12	96	24.2 (5.0)	71 (74.0)	55	24.9 (5.4)	33 (60.0)
2. McGlashan [60]	US	POPS	SIPS	Olanzapine (5–15 mg/day)	Placebo pill	12 months	6, 12	31	18.2 (5.5)	21 (67.7)	29	17.2 (4.0)	18 (62.1)
3. Woods [61]	US	SIPS	SIPS	Ziprasidone (20–160 mg/day) + Supportive Interpersonal Therapy	Placebo pill + Supportive Interpersonal Therapy	6 months	6	24	NR	NR	27	NR	NR
4. Yung [53]^c^, McGorry [54]^c^	Australia	CAARMS	CAARMS	Risperidone (max 2 mg/day) + CBT (CBT focussed on patients’ need, including subthreshold psychotic symptoms)	Placebo pill + CBT (CBT focussed on patients’ need, including subthreshold psychotic symptoms)	12 months	6, 12	43	17.6 (3.0)	15 (34.9)	44	18.0 (2.7)	17 (38.6)
Omega 3													
1. Amminger [62], Amminger [63, 70]	Austria	PANSS	PANSS	ω-3 PUFAs (1.2 g/day)	Placebo pill	3 months	6,12,36+	41	16.8 (2.4)	14 (34.0)	40	16.0 (1.7)	13 (33.0)
2. Cadenhead [64]	US	SIPS	SIPS	ω-3 PUFAs (740 mg EPA and 400 mg DHA/day)	Placebo pill	6 months	6,12	65			62		
3. McGorry [65], Nelson [66] (NEURAPRO)	Australia	CAARMS	CAARMS	ω-3 PUFAs (1.4 g/day) *0*–*6 months*. ω-3 PUFAs (1.4 g/day) + CBCM *6*–*12 months*.	Placebo pill *0*–*6 months*. Placebo pill + CBCM *6-12 months*.	12 months	6,12	153	19.4 (4.8)	78 (51.0)	151	18.9 (4.3)	61 (40.4)
4. Qurashi [71]^d^ (NAYAB)	Pakistan	CAARMS	CAARMS	ω-3 PUFAs (1.2 g/day; 720 mg EPA and 480 mg DHA/day)	Placebo pill	12 months	6, 12	80	23.8 (5.4)	47 (58.8)	82	23.9 (5.3)	50 (61.0)
Minocycline													
1. Qurashi [71]^d^ (NAYAB)	Pakistan	CAARMS	CAARMS	Minocycline [Minocycline + ω-3 PUFAs]^d^	Placebo pill	12 months	6, 12	82	25.2 (5.3)	48 (59.5)	82	23.9 (5.3)	50 (61.0)
Combined (psychotherapy + antipsychotics)													
1. McGorry [67], Phillips [69]	Australia	CAARMS	CAARMS	Risperidone (max 2 mg/day) + CBT	NBI (Needs-based psychotherapy focussed on social relationships and vocational/family issues + needs-based pharmacotherapy)	6 months	6, 12, 36–48	31	20 (4.0)	20 (65)	28	20 (3.0)	14 (50)
2. Yung [53]^b^, McGorry [54]^b^	Australia	CAARMS	CAARMS	Risperidone (max 2 mg/day) + CBT (CBT focussed on patients’ need, including subthreshold psychotic symptoms)	Supportive therapy + Placebo pill (“Therapy aimed to provide the patient with emotional and social support, basic problem solving, stress management, psychoeducation about psychosis”)	12 months	6, 12	43	17.6 (3.0)	15 (34.9)	28	18.8 (3.7)	13 (46.0)
Transcranial Magnetic Stimulation (TMS)													
1. Tang [30]	China	SIPS	SIPS	TMS over the parieto-hippocampal network (10 sessions over two days)	Sham TMS	2 days	12 months	31	19.0 (5.9)	12 (39.7)	27	19.6 (6.0)	19 (70.4)

*BPRS* brief psychiatric rating scale, *CAARMS* comprehensive assessment of the at-risk mental state, *CBT* cognitive behavioral therapy, *CBCM* cognitive behavioral case management, *COGDIS* basic symptoms criterion cognitive disturbance, *ERIraos* early recognition inventory, *F-ACT* family aided – Assertive Community Treatment, *FFT* family focused treatment, *GAF* global assessment of functioning, *IPI* integrated psychological intervention, *NBI* needs based intervention, *NDRL* non directive reflective listening, *NR* not reported, *POPS* presence of psychotic symptoms scale, *SANS* scale for the assessment of negative symptoms, *SFS* social functioning scale, *SST* social skill training, *TAU* treatment as usual, ω-3, *PUFAs* long-chain ω-3 polyunsaturated fatty acids.

^a^This is a three arms trial, here we report the comparison of the two arms that were used to obtain the meta-analytic estimates on the efficacy of psychotherapies.

^b^This is a three arms trial, here we report the comparison of the two arms that were used to obtain the meta-analytic estimates on the efficacy of combined therapy.

^c^This is a three arms trial, here we report the comparison of the two arms that were used to obtain the meta-analytic estimates on the efficacy of antipsychotics.

^d^This is a four arms trial, here we report the comparison of the two arms that were used to obtained the meta-analytic estimates on the efficacy of omega-3.

Table 1 reports a descriptive summary of the included RCTs.

### Transition from CHR-P state to FEP

Of the ten RCTs comparing CBT vs control interventions in CHR-P, nine reported data on transition to psychosis at 6 months [27–29, 47–49, 51–53]; nine reported data at 12 months [28, 29, 47–52, 54]; three at 18 months [47, 49, 52]; one at 24 months [49]; two at +36 months [68, 73]. One study was excluded from the analyses [50] due to high risk of bias [24]. The meta-analytic estimates showed that CBT was not superior to control interventions in reducing the risk of transition to FEP at 6 months (9 RCTs; OR: 0.84; 95% CI: 0.52–1.35; I^2^: 0%; *P* = 0.47) and at 12 months (9 RCTs; OR: 0.64; 95% CI: 0.39–1.06; I^2^: 21%; *P* = 0.08); it was effective at 18 months (3 RCTs; OR: 0.49; 95% CI: 0.27–0.90; I^2^: 0%; *P* = 0.02), but not at 24 months (only one RCT [49]) or at +36 months (2 RCTs; OR: 0.58; 95% CI: 0.31–1.07; I^2^: 0%; *P* = 0.08).

In the RCTs on cognitive remediation [58], sleep intervention [31], and ‘systemic therapy’ [57], the active intervention was not superior to the control intervention in reducing the risk of psychosis transition in CHR-P. The integrated psychotherapy approach [59] and the TMS intervention [30] reported a significant effect in reducing the risk of psychosis compared to the control condition. It was not possible to provide a quantitative summary for these interventions as only a single RCT per intervention was available. The two RCTs investigating family focused therapies reported mixed findings, with one RCT showing efficacy in reducing transition risk [56] and the other not [55]. We did not provide a quantitative summary for family-focused therapies, as data on psychosis transitions were provided only at largely distant time points (6 months [57] and +36 months [55]); also the treatment approach was different despite the similar labeling (see Table 1).

Of the four RCTs that compared antipsychotics vs placebo [28, 53, 54, 60, 61], all reported data on transition to psychosis in CHR-P at 6 months, three reported data at 12 months [28, 53, 54, 60], and none at 18, 24, 36, and +36 months. Among these four RCTs, one used olanzapine 5–15 mg/day as active treatment [60], one used Aripiprazole 5–15 mg/day [28], one Ziprasidone 20–160 mg/day [61], and one Risperidone 2 mg/day [53, 54]. The meta-analytic estimates at 6 (OR: 0.63; 95% CI: 0.31–1.27; I^2^: 0%; *P* = 0.20) and 12 months (OR: 0.87; 95% CI: 0.32–2.32; I^2^: 57%; *P* = 0.78) showed that antipsychotics were not superior to placebo in reducing the rates of transition to psychosis in CHR-P.

The two RCTs that compared CBT plus risperidone to supportive therapy plus placebo pills reported data at 6 and 12 months [53, 54, 67, 69]; one of these two RCTs reported data at 18 months [67, 69]. The meta-analytic estimates showed that CBT plus risperidone was superior to the control intervention in reducing the risk of transition to psychosis in CHR-P at 6 months (OR: 0.29, 95% CI: 0.09–0.91; I^2^: 0%; *P* = 0.03), but not at 12 months (OR: 0.55, 95% CI: 0.24–1.28; I^2^: 0%; *P* = 0.17); null findings were also reported by the only RCT that collected data at 18 months [67, 69].

All four RCTs that compared omega-3 fatty acids to placebo pills [62, 64–66, 70, 71] reported data on transition to psychosis in CHR-P at 6 and 12 months; none at 18, 24, 36 months; two at +36 months [66, 70]. The meta-analytic estimates showed that omega-3 fatty acids were not superior to placebo in reducing risk of psychosis transition in CHR-P at either 6 (OR: 0.93, 95% CI: 0.23–3.76; I^2^: 66%; *P* = 0.91), 12 (OR: 0.72, 95% CI: 0.14–3.69; I^2^: 71%; *P* = 0.69), or +36 months (OR: 0.41, 95% CI: 0.08–2.12; I^2^: 83%; *P* = 0.29).

One (RCT) investigated the effects of minocycline, a combination of minocycline and omega-3 fatty acids, and placebo pills on psychosis transition [71]. The study found no significant difference in psychosis transition rates among the three groups.

### Secondary outcomes

The meta-analytic estimates on acceptability of preventative interventions in CHR-P did not show any significant difference for any of the active interventions when compared to the respective control interventions. Similarly, the meta-analytic estimates on attenuated psychotic symptoms, negative symptoms, and functioning did not show any significant difference at any of the investigated time points between any of the active and control interventions. These results are reported in Supplementary material (eTable2)

### Publication biases and risk of bias

No publication bias was detected with funnel plots. Results from the risk of bias tool are presented in Supplementary material (eTable1) Only one study was considered at high risk of bias [50] due to issues related to the randomization process, outcome measures, and selection of reported results.

## Discussion

In this paper, we presented data of an updated systematic review and meta-analysis on preventative interventions in CHR-P. We summarized data from twenty-four RCTs, providing a quantitative summary on the efficacy and acceptability of CBT, omega-3 fatty acids, antipsychotics, and CBT plus antipsychotics. We also provided a qualitative summary of other non-pharmacological and pharmacological approaches (family therapy, ‘systemic therapy’, sleep-targeted therapy, cognitive remediation, TMS, minocycline, minocycline plus omega-3).

Compared to the most recent meta-analyses in this field [25, 74, 75], we included new data from three of the largest RCTs ever conducted on CBT [27–29]. These RCTs included 764 CHR-P: adding these studies, almost doubled the total number of CHR-P available for meta-analysis of CBT trials in this group (previously, *N* = 830) [74]. Furthermore, we included in our qualitative synthesis three RCTs investigating innovative preventive approach in CHR-P, i.e., sleep therapy [31], TMS [30], and minocycline [71].

Our main finding was that CBT was not more effective than the control interventions in preventing transition to FEP in CHR-P at both 6 and 12 months. This finding updates the last meta-analyses in the field that reported positive findings [74, 75]. The discrepancy is attributable to the inclusion of three subsequently RCTs investigating CBT in CHR-P, all of which found no difference in psychosis transition risk compared to a control intervention. Although we found that the effect of CBT was superior to control interventions in at 18 months, the three most recently published RCTs did not collect data at this timepoint [27–29]. As these large RCTs did not show superiority to CBT over control interventions in preventing psychosis transition in CHR-P, the pooled estimate based on 18 months data cannot be interpreted as evidence of efficacy.

Moreover, RCTs that reported outcomes at timepoints later than 18 months (i.e., 24 [49] and +36 months [68]) showed no difference in psychosis transition between CBT and a control intervention.

Our quantitative summary on Omega-3 fatty acids, antipsychotics, and CBT plus antipsychotics confirmed previous evidence on lack of efficacy of these interventions in preventing psychosis in CHR-P at any of the investigated time points [25]; negative findings were also reported by the individual trials investigating cognitive remediation [58], a new sleep-targeted intervention [31], and ‘systemic therapy’[57]. These results are in line with the meta-analytic estimates on our secondary outcomes, showing no effect of any of the investigated interventions on symptom severity (positive and negative symptoms) and functioning at any of the investigated time points.

The most promising results on reducing risk of psychosis transition in CHR-P were reported by the only trial investigating an integrated psychological approach, combining CBT, cognitive remediation, and psychoeducation [59]. The only trial investigating brain stimulation in CHR-P reported a significant effect of TMS over sham in preventing psychosis at 12 months when compared to sham [30]. However the sample size was small and the stimulation protocol included only 2 sessions, therefore replication is needed.

### Future directions

1. ‘The absence of evidence does not equate to evidence of absence’ [76].
  Over the past decades, clinical early detection services for people at CHR-P have progressively gained more funding, personnel, and expertise. As a result of this, the standard of what is considered now “case management” or ‘needs base care’ can be quite high. Over the course of the years, we often observed successful clinical trials [47, 62] that failed to replicate initial findings [27, 65]. It is possible that this might be the consequence of increasingly more effective case management producing a ceiling effect [28]. If this is the case, this would suggest that case management might represent a preventative treatment in its own right. Future trials could address this by using a multi-arm, multistage (MAMS) design, where case management is directly compared to a number of active interventions.
2. Addressing heterogeneity in CHR-P: a call for precision preventative psychiatry.
  Our meta-analytic estimates presented with substantial heterogeneity. Some effective signal in treatments’ efficacy may have been missed because of the heterogeneity of the population. However, we previously simulated a living meta-analysis [77], which showed that the current CBT trials have adequate power (90%) to detect a 50% risk ratio reduction in transition to psychosis. These findings indicate that even the publication of future CBT RCTs in this population are unlikely to change the level of evidence. This is empirically consistent with the observation that the largest RCTs to date are all negative and do not support the efficacy of CBT for psychosis prevention [27, 28].
  Future clinical trials could benefit from implementing treatment stratification strategies, with intervention targeted at subgroups that are most likely to respond. However, this approach entails the initial recruitment of relatively large participant samples, and therefore requires large scale trials involving multiple centres. Ongoing programmes that have recently adopted this approach include the STEP trial (https://www.theguardian.com/society/2023/feb/16/global-oxford-study-trial-cannabis-based-medicine-treatment-psychosis-cbd) and the Accelerating Medicine Programme for Schizophrenia (AMP-SCZ).
3. The need for novel treatment approaches.
  Our findings indicate the need for a new generation of preventive interventions for psychosis in CHR-P. There is a consensus that in addition to efficacy, preventive treatments for this group should also be well-tolerated and non-stigmatising. This is a particular issue for pharmacological interventions, but some compounds, such as CBD and oxytocin, have a relatively high patient acceptability as well as evidence for effectiveness [77, 78]. Psychotic disorders are highly heterogeneous at any stage of illness [79]. Even well-established treatments for psychotic disorders, such as antipsychotic medications, are not effective for all patients with a confirmed diagnosis [79]. As prognostic uncertainty is even larger in CHR-P patients, any future trial will need sufficient power to detect a significant effect and identify those patients that benefit most from it. Promising non pharmacological interventions include the integrated psychological intervention developed by Bechdolf et al. [59].
4. Focusing on psychosis transition as a main outcome.

Prevention of psychosis should remain the cornerstone and the most important outcome for the CHR-P field, complemented by other outcomes (e.g., functioning [29], physical health [80], distress from symptoms and sleep [31]). Current claims that prevention of psychosis is a second-order issue could discourage future trials from exploring this relevant outcome and the current uncertainty in the meta-analytical estimates may remain unresolved.

#### Strengths and limitations

A key strength of this meta-analysis is that the numbers of CHR-P included in the analysis of our primary outcome is almost doubled that in previous works. Moreover, we investigated the efficacy of specific preventive interventions without lumping together diverse approaches, which would not be clinically informative.

The measure of our primary outcome was based on transition criteria as defined by individual studies (see Table 1). This might have resulted in increased heterogeneity, although the use of a random effects model should have partially addressed this issue. Because most of the trials in the literature have not reported outcome data beyond 12 months, we were unable to fully assess effectiveness in the longer term. Finally, while most trials reported dropout due to any cause, this is a relatively crude measure of treatment acceptability. A more direct measure, such as specific adverse effects, may have revealed significant differences between treatments, especially for trials of antipsychotic medications. However, these outcomes are rarely reported in the CHR-P literature.

## Supplementary information

Supplement
