# Supplementary material for: Preventing psychosis in people at clinical high risk: an updated meta-analysis by the World Psychiatric Association Preventive Psychiatry section
Source: Mol Psychiatry. 2025 Feb 14;30(6):2773–82. doi: 10.1038/s41380-025-02902-8 (PMC12092282; doi:10.1038/s41380-025-02902-8)
Supplement: Supplementary file 1 — Supplement [file 41380_2025_2902_MOESM1_ESM.docx]

**Supplementary Online Content**

Table of Contents

[Assessing the Methodological Quality of Systematic Reviews 2](#_Toc149909956)

[List of changes from the original protocol 5](#_Toc149909957)

[eTable 1. Risk of bias summary table 6](#_Toc149909958)

[eTable2. Meta-analytic estimates for secondary outcomes at different time points 7](#_Toc149909959)

# **Assessing the Methodological Quality of Systematic Reviews**

**
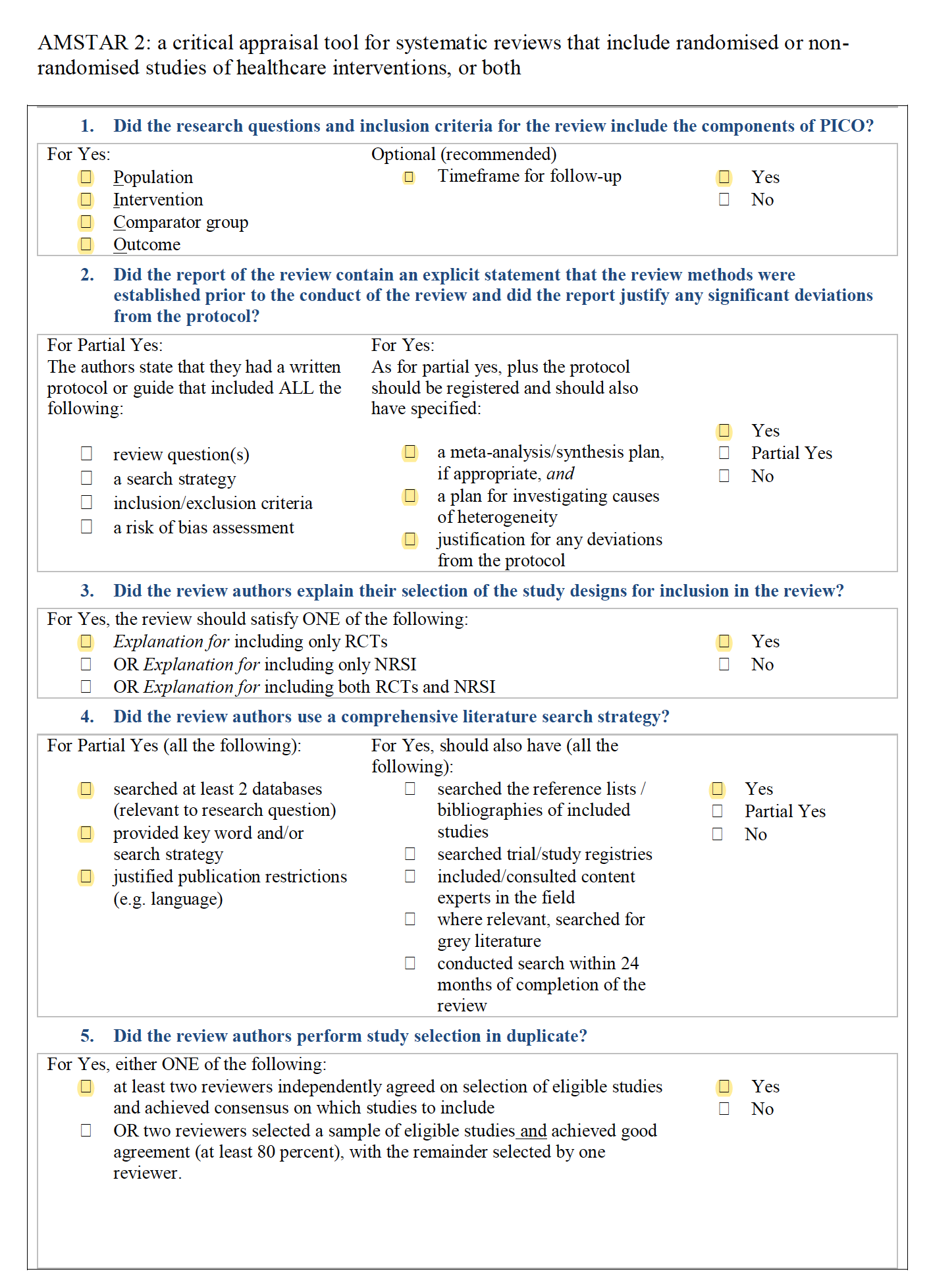
**

**
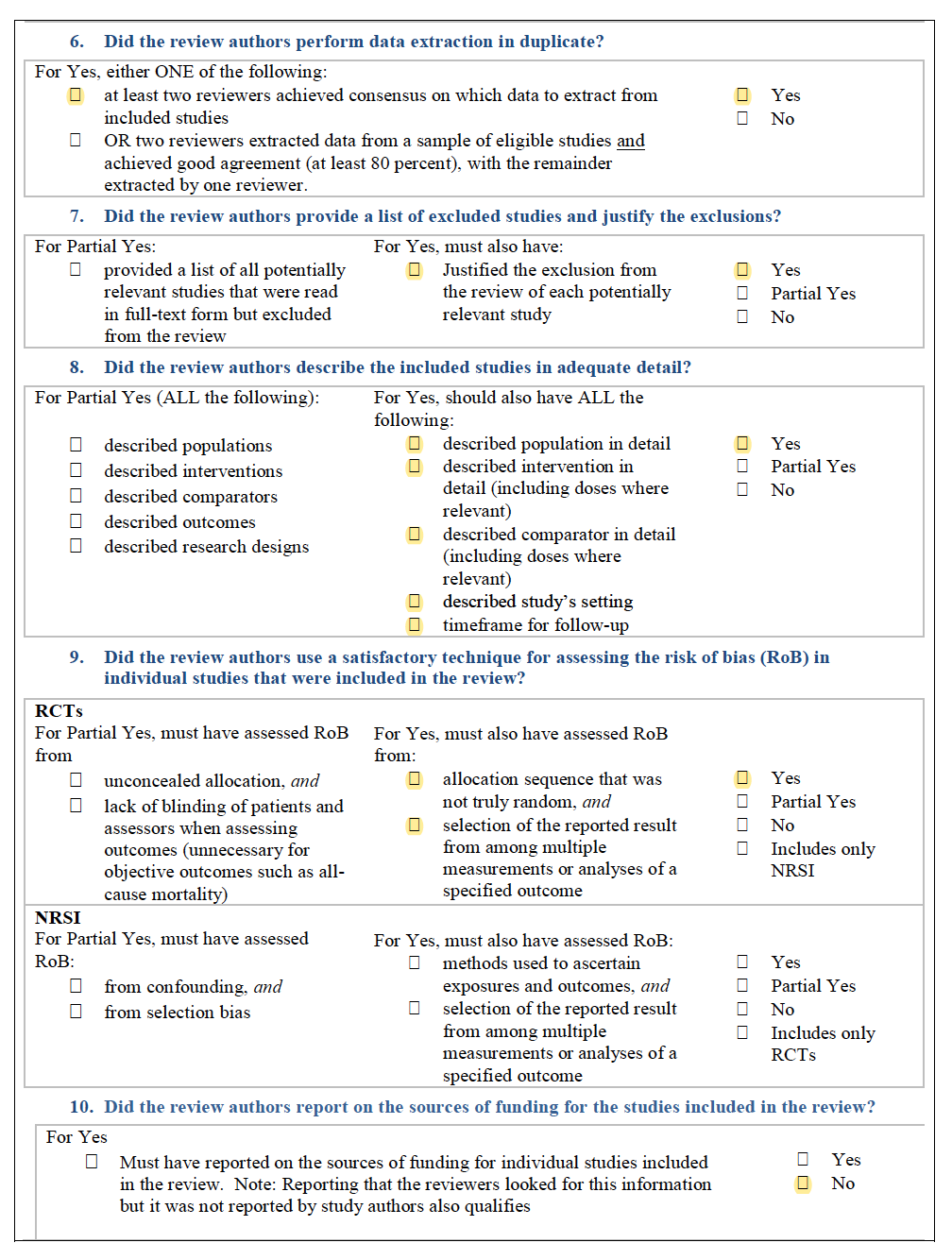
**

**
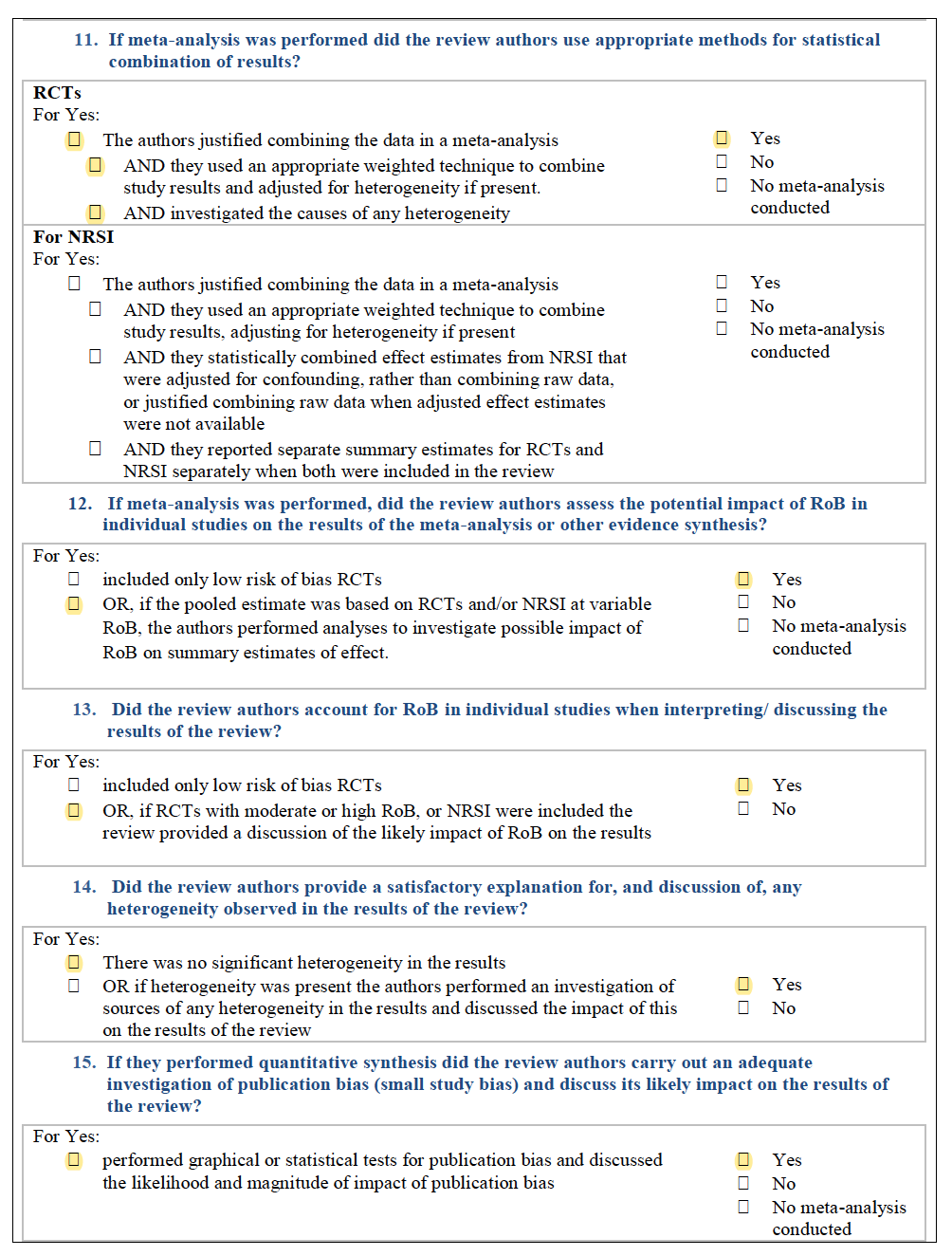
**

**
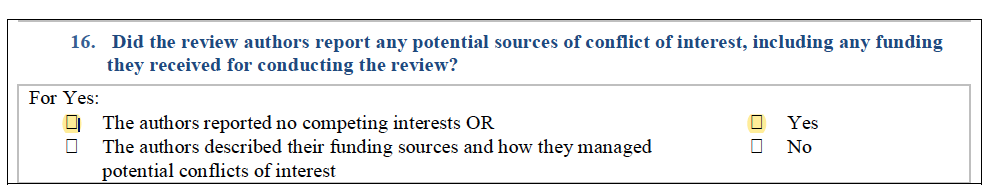
**

# **List of changes from the original protocol**

1. In the published protocol of this study (DOI 10.17605/OSF.IO/EUSKB), the original plan was to conduct the analysis by lumping together all psychosocial and psychotherapy interventions. However, in order to provide precise recommendations, we soon realised that we needed to consider specific therapeutic approaches as individual categories of intervention. This was particularly important for CBT, as three new RCTs were published after the publication of our protocol.
2. In the published protocol of this study, the original plan was to conduct the analysis by including also studies at high risk of bias and then perform sensitivity analyses by excluding them. However, as only one small RCT was at high risk of bias, we decided to analyse and present the results by directly excluding it.

# **eTable 1. Risk of bias summary table**

|  | **Randomization process** | **Deviation from intended interventions**  *(Effect to assignment to intervention)* | **Deviation from intended interventions**  *(Effect to adhering to intervention)* | **Missing outcome data** | **Measurement of the outcome** | **Selection of reported results** | **Overall risk of bias** |
| --- | --- | --- | --- | --- | --- | --- | --- |
| **CBT** |  |  |  |  |  |  |  |
| Addington 2011 | - | - | - | - | - | - | - |
| Addington 2023 | - | - | - | - | - | - | - |
| Bechdolf 2023 | - | - | - | - | - | - | - |
| McGorry 2023 | - | - | - | - | - | - | - |
| Morrison 2004 | - | - | - | - | - | - | - |
| Morrison 2012 | - | - | - | - | - | - | - |
| Pozza 2020 | - | - | - | - | - | - | + |
| Van der Gaag 2012 | - | - | - | - | - | - | - |
| Stain 2016 | - | - | - | - | - | - | - |
| Yung 2011 | - | - | - | - | - | - | - |

Legend. In green: Low risk of bias; In yellow: some concerns; In red: High risk of bias.
Please note that RoB2 was performed only on CBT interventions as, in line with Cochrane Handbook, the pooled estimates were significant only for this category of interventions. Studies were considered at high-risk bias when at least one domain was at high risk of bias or three or more domains were presenting some concerns

# **eTable2**. Meta-analytic estimates for secondary outcomes at different time points

| **Acceptability** | | | | | | |
| --- | --- | --- | --- | --- | --- | --- |
| ***Active intervention*** | ***Time point*** | ***RCTs*** | ***Odd Ratio*** | ***95%CI*** | ***I^2^*** | ***P*** |
| CBT | End of treatment | N=9 [1-9] | 0.96 | 0.76 to 1.22 | 0% | 0.75 |
| FFT | End of  Treatment | N=2 [10, 11] | 0.63 | 0.33 to 1.21 | 0% | 0.16 |
| Omega 3 | End of treatment | N=3 [12-14] | 0.84 | 0.52 to 1.36 | 0% | 0.45 |
| Antipsychotics | End of treatment | N=3 [3, 8, 15] | 0.97 | 0.60 to 1.57 | 0% | 0.91 |
| CBT + Risperidone | End of treatment | N=2 [8, 16] | 1.25 | 0.46 to 3.42 | 0% | 0.66 |
| **Attenuated Psychotic symptoms^1^** | | | | | | |
| ***Active intervention*** | ***Time point*** | ***RCTs*** | ***SMD*** | ***95%CI*** | ***I^2^*** | ***P*** |
| CBT | 6 months | N=8 [1-8] | -0.35 | -1.03 to 0.34 | 96% | 0.32 |
|  | 12 months | N=8 [1, 2, 5-9] | -0.35 | -0.80 to 0.13 | 89% | 0.13 |
|  | 18 months | N=3 [2, 5, 7] | -0.18 | -0.42 to 0.07 | 0% | 0.16 |
| Omega 3 | 6 months | N=3 [12-14] | -0.25 | -0.61 to 0.10 | 72% | 0.17 |
|  | 12 months | N=3 [12-14] | -0.14 | -0.44 to 0.16 | 58% | 0.36 |
|  | +36 months | N=2 [12, 13] | -0.31 | -1.13 to 0.51 | 89% | 0.46 |
| Antipsychotics | 6 months | N=3 [3, 8, 17] | -0.21 | -1.17 to 0.75 | 90% | 0.66 |
|  | 12 months | N=3 [3, 8, 17] | -0.21 | -0.45 to 0.02 | 0% | 0.07 |
| CBT + Risperidone | 6 months | N=2 [8, 16] | 0.02 | -0.33 to 0.37 | 0% | 0.92 |
|  | 12 months | N=2 [8, 16] | 0.00 | -0.38 to 0.38 | 16% | 1.00 |
| **Negative Symptoms^2^** | | | | | | |
| ***Active intervention*** | ***Time point*** | ***RCTs*** | ***SMD*** | ***95%CI*** | ***I^2^*** | ***P*** |
| CBT | 6 months | N=5 [1-4, 8] | -0.29 | -1.02 to 0.43 | 94% | 0.43 |
|  | 12 months | N=4 [1-3, 8] | -0.32 | -1.22 to 0.58 | 95% | 0.49 |
| Omega 3 | 6 months | N=2 [12, 13] | -0.14 | -0.60 to 0.32 | 71% | 0.55 |
|  | 12 months | N=8 [1, 2, 5-9] | -0.21 | -0.50 to 0.08 | 38% | 0.15 |
| Omega 3 | +36 months | N=2 [12, 13] | -0.25 | -0.70 to 0.20 | 63% | 0.28 |
| Antipsychotics | 12 months | N=3 [3, 15] | 0.27 | -0.10 to 0.65 | 60% | 0.16 |
| CBT + Risperidone | 6 months | N=2 [8, 16] | 0.13 | -0.68 to 0.94 | 81% | 0.76 |
|  | 12 months | N=2 [8, 16] | 0.12 | -0.23 to 0.47 | 0% | 0.49 |
| **Functioning^3^** | | | | | | |
| ***Active intervention*** | ***Time point*** | ***RCTs*** | ***SMD*** | ***95%CI*** | ***I^2^*** | ***P*** |
| CBT | 6 months | N=8 [1-8] | 0.11 | -0.26 to 0.49 | 87% | 0.55 |
|  | 12 months | N=7 [1-3, 5-8] | 0.20 | -0.10 to 0.49 | 73% | 0.19 |
|  | 18 months | N=3 [2, 5, 7] | 0.23 | -0.02 to 0.48 | 0% | 0.07 |
| Omega 3 | 6 months | N=2 [12, 13] | 0.20 | -0.69 to 1.08 | 92% | 0.32 |
|  | 12 months | N=2 [12, 13] | 0.41 | -0.39 to 1.21 | 90% | 0.32 |
|  | +36 months | N=2 [12, 13] | 0.12 | -0.51 to 0.76 | 82% | 0.70 |
| Antipsychotics | 12 months | N=3 [3, 8, 17] | -0.06 | -0.29 to 0.17 | 0% | 0.60 |
| CBT + Risperidone | 12 months | N=2 [8, 16] | 0.01 | -0.34 to 0.36 | 0% | 0.96 |

In case individual studies used multiple measures of APS we extracted data from the clinical rating scale that was used as CHR-P ascertainment method.

2 In case individual studies used multiple measures of negative symptoms we extracted data from the SANS (primary measure); if the SANS was not available, we extracted data from other scales in the following order of preference: PANSS, BPRS, CAARMS/SIPS, BIPS, ERIraos;
^3^In case individual studies used multiple measures of functioning, we extracted data from the SOFAS (primary measure), if the SOFAS was not available we extracted data from other scales in following order of preference: GAF, combined score of GF:S and GF:R.

**References**

1. Addington, J., et al., *Cognitive-Behavioral Social Skills Training: Outcome of a Randomized Controlled Trial for Youth at Risk of Psychosis.* Schizophr Bull Open, 2023. **4**(1): p. sgad020.

2. Addington, J., et al., *A randomized controlled trial of cognitive behavioral therapy for individuals at clinical high risk of psychosis.* Schizophr Res, 2011. **125**(1): p. 54-61.

3. Bechdolf, A., et al., *Prevention of First-Episode Psychosis in People at Clinical High Risk: A Randomized Controlled, Multicentre Trial Comparing Cognitive-Behavioral Therapy and Clinical Management Plus Low-Dose Aripiprazole or Placebo (PREVENT).* Schizophr Bull, 2023. **49**(4): p. 1055-1066.

4. McGorry, P.D., et al., *A Sequential Adaptive Intervention Strategy Targeting Remission and Functional Recovery in Young People at Ultrahigh Risk of Psychosis: The Staged Treatment in Early Psychosis (STEP) Sequential Multiple Assignment Randomized Trial.* JAMA Psychiatry, 2023. **80**(9): p. 875-885.

5. Morrison, A.P., et al., *Early detection and intervention evaluation for people at risk of psychosis: multisite randomised controlled trial.* BMJ, 2012. **344**: p. e2233.

6. Stain, H.J., et al., *A randomised controlled trial of cognitive behaviour therapy versus non-directive reflective listening for young people at ultra high risk of developing psychosis: The detection and evaluation of psychological therapy (DEPTh) trial.* Schizophr Res, 2016. **176**(2-3): p. 212-219.

7. van der Gaag, M., et al., *Cognitive behavioral therapy for subjects at ultrahigh risk for developing psychosis: a randomized controlled clinical trial.* Schizophr Bull, 2012. **38**(6): p. 1180-8.

8. Yung, A.R., et al., *Randomized controlled trial of interventions for young people at ultra high risk for psychosis: 6-month analysis.* J Clin Psychiatry, 2011. **72**(4): p. 430-40.

9. Morrison, A.P., et al., *Cognitive therapy for the prevention of psychosis in people at ultra-high risk: randomised controlled trial.* Br J Psychiatry, 2004. **185**: p. 291-7.

10. W, M., *Early Detection, Intervention and Prevention (EDIP).* NCT01597141, 2016.

11. Miklowitz, D.J., et al., *Family-focused treatment for adolescents and young adults at high risk for psychosis: results of a randomized trial.* J Am Acad Child Adolesc Psychiatry, 2014. **53**(8): p. 848-58.

12. McGorry, P.D., et al., *Effect of omega-3 Polyunsaturated Fatty Acids in Young People at Ultrahigh Risk for Psychotic Disorders: The NEURAPRO Randomized Clinical Trial.* JAMA Psychiatry, 2017. **74**(1): p. 19-27.

13. Amminger, G.P., et al., *Long-chain omega-3 fatty acids for indicated prevention of psychotic disorders: a randomized, placebo-controlled trial.* Arch Gen Psychiatry, 2010. **67**(2): p. 146-54.

14. Qurashi, I., et al., *A randomised double-blind placebo-controlled trial of minocycline and/or omega-3 fatty acids added to treatment as usual for at risk Mental States: The NAYAB study.* Brain Behav Immun, 2023. **115**: p. 609-616.

15. McGlashan, T.H., et al., *Randomized, double-blind trial of olanzapine versus placebo in patients prodromally symptomatic for psychosis.* Am J Psychiatry, 2006. **163**(5): p. 790-9.

16. McGorry, P.D., et al., *Randomized controlled trial of interventions designed to reduce the risk of progression to first-episode psychosis in a clinical sample with subthreshold symptoms.* Arch Gen Psychiatry, 2002. **59**(10): p. 921-8.

17. Woods S, S.J., Compton M, Daley M, Rajarethinam R, Graham K, Breitborde N, Cahill J, Srihari V, Perkins D, Bearden C, Cannon T, Walker E, McGlashan T. , *Effects of Ziprasidone Versus Placebo in Patients at Clinical High Risk for Psychosis.* Schizophr Bull, 2017. **Mar;43(Suppl 1):S58.**
